# Culturable yeasts associated to different life stages of the farmed red seaweed *Porphyra dioica*

**DOI:** 10.3389/fmicb.2026.1918765

**Published:** 2026-08-12

**Authors:** Mónica A. Fernandes, Madalena Matos, Tina Keller-Costa, Rodrigo Costa, Madalena C. Mendes, Ana Coutinho, Inês Oliveira, Margarida Martins, Isabel Sá-Correia

**Affiliations:** 1 Institute for Bioengineering and Biosciences (iBB), Instituto Superior Técnico, Universidade de Lisboa, Lisbon, Portugal; 2 Associate Laboratory i4HB-Institute for Health and Bioeconomy at Instituto Superior Técnico, Universidade de Lisboa, Lisbon, Portugal; 3 Department of Bioengineering, Instituto Superior Técnico, Universidade de Lisboa, Lisbon, Portugal; 4 PCI–Creative Science Park, ALGAplus-Production and Trading of Seaweed and Derived Products S.A., Ílhavo, Portugal; 5 GreenCoLab, Associação Oceano Verde, Universidade do Algarve, Faro, Portugal; 6 Department of Chemistry, Centre for Environmental and Marine Studies, University of Aveiro, Aveiro, Portugal

**Keywords:** algal-associated yeasts, biotechnological potential, culturable marine yeasts, farmed algae, IMTA system, life stage distribution, yeast diversity

## Abstract

Macroalgal-associated microbiomes are increasingly recognized as key determinants of host fitness, biogeochemical cycling, and cultivation stability in industrial aquaculture. While bacterial assemblages of red macroalgae have received considerable attention, the associated mycobiome, and in particular the yeast fraction, remains poorly characterized, despite mounting evidence that fungal taxa contribute to phytohormone signaling, surface colonization dynamics, and host stress tolerance. In this study, we characterized the culturable yeast fraction associated with five life stages of the red macroalga *Porphyra dioica* cultivated in an Integrated Multi-Trophic Aquaculture (IMTA) system in Ílhavo, Portugal. Yeast isolates were recovered from conchocelis, conchosporangia, young blade, and adult blade stages by direct plating and enrichment on selective media and taxonomically assigned by rRNA gene sequencing. Isolates were further screened for functional traits of ecological and biotechnological relevance, including auxin biosynthesis, biosurfactant and bioemulsifier production, and intracellular lipid accumulation. A total of 21 isolates were recovered and assigned to *Rhodotorula mucilaginosa*, *Naganishia diffluens*, *Williopsis vartiovaarae*, *Taphrina* sp., and *Sporobolomyces roseus*, spanning both Ascomycota and Basidiomycota. Yeast recovery was constrained by chloramphenicol-resistant bacterial overgrowth in algae samples cultivated with filtered, non-sterilized seawater, and therefore the dataset represents the recoverable culturable fraction. Assemblages were structured by host developmental stage: *N. diffluens* was recovered from conchocelis, *R. mucilaginosa* from conchosporangia, and a shift from ascomycetous taxa (*W. vartiovaarae*, *Taphrina* sp.) to pigmented basidiomycetous yeasts (*N. diffluens*, *S. roseus*) was observed in adult blades approaching harvest. Several isolates produced auxin, biosurfactants, and bioemulsifiers, and accumulated intracellular lipids, supporting potential roles as probiotics in industrial macroalgal cultivation and as candidate strains for value-added bioproducts. This study provides a first insight into the culturable yeast community associated with industrially cultivated *P. dioica* and establishes a basis for future studies addressing the role of yeasts in macroalgal performance cultivated under IMTA systems.

## 1 Introduction

Macroalgae are photosynthetic, aquatic eukaryotes that play central ecological roles as primary producers, habitat-forming organisms and modulators of nutrient cycling. They also have increasing economic value as sources of food, feed, fertilizers, cosmetics, nutraceuticals and bioactive compounds (Araújo et al., 2021; Zhang et al., 2022). Macroalgae aquaculture is regarded as a sustainable source of biomass because it does not require arable land, freshwater irrigation and may contribute to nutrient bioremediation in circular aquaculture systems (Araújo et al., 2021; Hossain et al., 2022; Zhang et al., 2022). However, only a limited number of macroalgal species have stable and scalable cultivation protocols, and production can be constrained by environmental variability, disease, reproductive bottlenecks and incomplete control of early developmental stages (Ghaderiardakani et al., 2020; Mendes et al., 2022).

The associated microbiome is increasingly recognized as a key factor influencing macroalgal health and performance. Macroalgae live as holobionts in association with bacteria, archaea, fungi, yeasts, protists and viruses, which may contribute to nutrient acquisition, morphogenesis, settlement, stress tolerance, pathogen defense and acclimation to environmental change (Egan et al., 2013; Hollants et al., 2013; Singh and Reddy, 2015; Lian et al., 2018; Vila Duplá, 2025). Bacteria are the best-studied component of macroalgal microbiomes and can produce vitamins, siderophores, quorum-sensing molecules and morphogenetic factors involved in algal growth, spore germination, thallus development and disease resistance (Croft et al., 2005; Matsuo et al., 2005; Joint et al., 2007; Spoerner et al., 2012; Egan et al., 2013; Ramanan et al., 2016; Cortez et al., 2026). By contrast, the fungal fraction remains comparatively underexplored, particularly yeasts, which have mostly been addressed through isolation studies or broader surveys of fungal diversity (Loque et al., 2010; Duarte et al., 2016; Vallet et al., 2018).

Recent evidence supports the view that yeasts are functionally relevant members of aquatic photosynthetic systems rather than passive contaminants (Sá-Correia et al., 2026). In microalgal laboratory and industrial cultures, yeasts and yeast-like fungi may influence nutrient availability, organic matter turnover, aggregation, oxidative stress responses and culture stability (Sá-Correia et al., 2026). Moreover, yeasts isolated from industrial microalgal cultures can produce metabolites with ecological and biotechnological relevance, including lipids, auxins, biosurfactants, bioemulsifiers and pigments (Matos et al., 2025a,b; Sá-Correia et al., 2026). Nevertheless, their roles in macroalgal holobionts remain poorly understood, especially across different host developmental stages.

*Porphyra dioica* Brodie and Irvine, 1977 is a red macroalga belonging to the Bangiales, an order that includes some of the most economically important edible seaweeds, widely used as nori and nori-like products. *P. dioica* is valued for its nutritional composition, including high protein content, dietary fiber, vitamins, minerals and low but nutritionally relevant lipid fractions, particularly polyunsaturated fatty acids (Pereira and Yarish, 2010; Wells et al., 2017). Its heteromorphic life cycle alternates between a haploid gametophytic blade and a diploid sporophytic conchocelis phase (Candia et al., 1999; Pereira and Yarish, 2010). This heteromorphic life cycle poses challenges for aquaculture because each developmental stage has distinct physiological requirements and may differ in susceptibility to environmental stress, microbial colonization and disease.

Macroalgal microbiomes vary with host species, tissue type, season, geography, environmental conditions and developmental stage (Hollants et al., 2013; Ghaderiardakani et al., 2020; Davis et al., 2023; Cortez et al., 2026). In algae from the Bangiales order, the marked differences between gametophytic and sporophytic phases in morphology, physiology, surface properties and cultivation conditions are expected to influence microbial recruitment and persistence (Mikami and Takahashi, 2023). Stage-specific microbiomes may therefore affect critical transitions such as spore germination, conchocelis growth, blade formation and nursery performance (Pereira and Figueroa, 2025). However, it remains largely unknown whether yeasts are consistently associated with specific stages of *P. dioica*, whether their diversity changes during the life cycle, and whether isolates from different stages display traits relevant to host development or aquaculture performance.

Among the traits potentially involved in yeast-alga interactions, biosurfactant, bioemulsifier, auxin and lipid production are of particular interest. Biosurfactants and bioemulsifiers can increase the bioavailability of hydrophobic substrates, influence biofilm architecture, regulate microbial attachment and detachment, and contribute to antagonistic interactions within microbial communities (Ron and Rosenberg, 2001). Auxins regulate cell division, elongation and differentiation in land plants, and auxin-like effects have also been reported in algae, including red macroalgae, where moderate concentrations may influence growth and development (Lin and Stekoll, 2007; Stirk et al., 2013; Cheng et al., 2023). Lipid accumulation by yeasts is also relevant to carbon storage and turnover and may provide high-value compounds (Mota et al., 2022). These traits provide a useful framework for assessing whether culturable yeasts associated with macroalgae have functional potential within the holobiont.

The present study aimed to characterize the culturable yeast community associated with different life-cycle stages of *Porphyra dioica* cultivated at ALGAplus, Ílhavo, Portugal, in a land-based Integrated Multi-Trophic Aquaculture (IMTA) system. In this system, water from higher trophic-level production units is used to cultivate lower trophic-level organisms, promoting nutrient recycling and reducing environmental impacts without the use of artificial pesticides or fertilizers (Hossain et al., 2022; Pereira et al., 2025). Yeasts were isolated from distinct developmental stages of *P. dioica*, identified, and screened for traits with potential ecological and biotechnological relevance, including lipid accumulation, biosurfactant and bioemulsifier production, auxin production and growth on alternative carbon sources. By linking yeast diversity with algal developmental stage and functional potential, this work addresses an overlooked component of the *P. dioica* microbiome and contributes to a broader understanding of yeast roles in cultivated marine algal systems.

## 2 Materials and methods

### 2.1 *Porphyra dioica* culture sampling for yeast isolation

**Figure 1:**
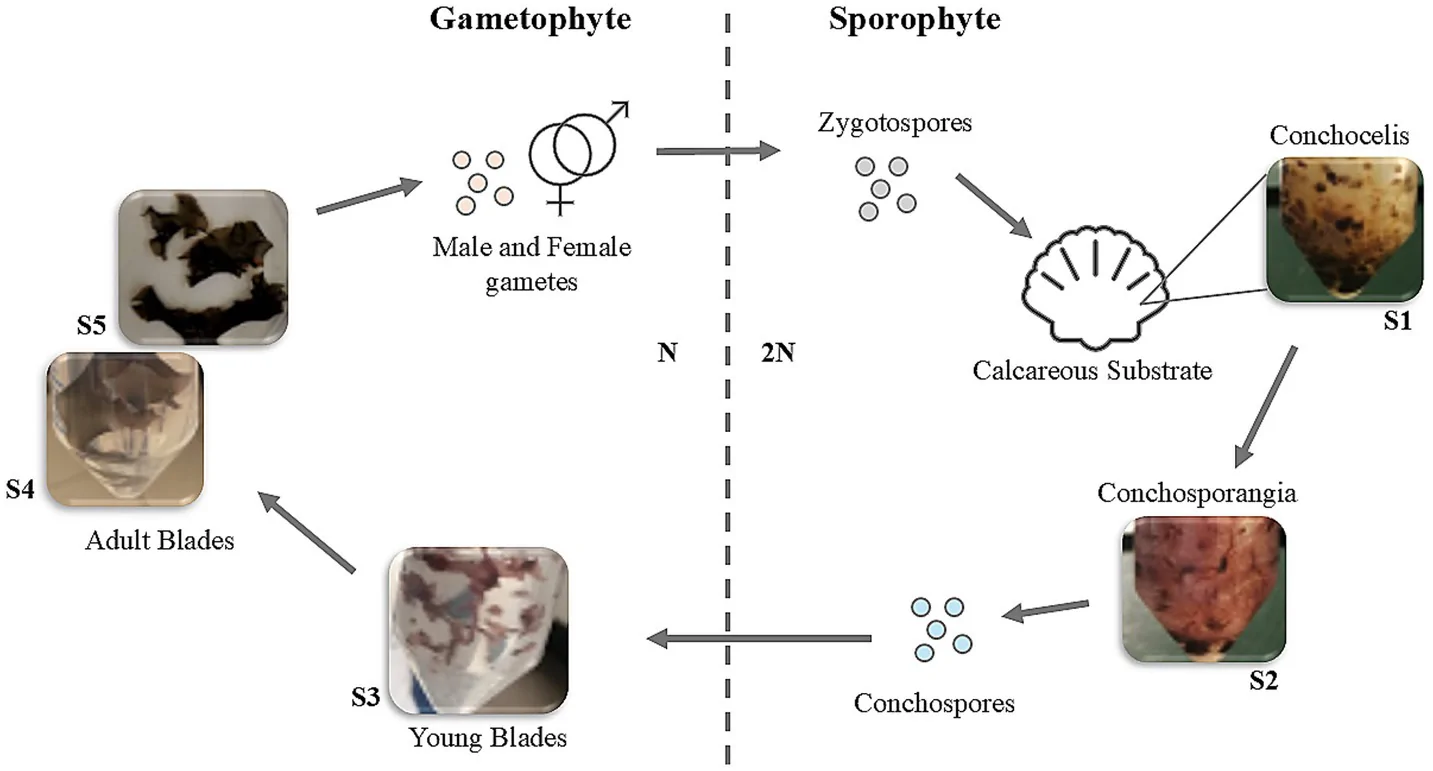
Life cycle of *Porphyra dioica* with photographic evidence of the five life stages (S1 to S5) sampled for this study.

Seaweed samples from the species *Porphyra dioica* were farmed, collected (on the 9th of November 2023) and provided by ALGAplus (https://www.algaplus.pt/, Ílhavo, Aveiro, Portugal). ALGAplus farms organic certified seaweed including *P. dioica*, using a land-based Integrated Multi-Trophic Aquaculture (IMTA) system where nutrient-rich water from the organic fish production (sea bream and sea bass) is used as resource to cultivate seaweed, without using artificial fertilizers. *P. dioica* cultivation begins in a nursery, designed to support the algae during their early stages of growth. Next, algae are transferred to outdoor cultivation tanks, where they grow until they are ready to be harvested. Due to operational constraints associated with the production cycle, it was not feasible to obtain multiple biological replicates per stage. The five collected samples, one sample for each life cycle, (Figure 1) correspond to biological material from five different life stages of *Porphyra dioica* (S1, S2, S3, S4, and S5) and respective culture water.

Briefly, S1 corresponds to mother cultures of conchocelis growing vegetatively, S2 corresponds to the conchosporangia stage, S3 corresponds to vegetatively growing young blades. S1, S2 and S3 stages were grown indoors in small photobioreactors (250 mL–20 L) and cultured in batch conditions under controlled stocking density, temperature, light, and aeration conditions, with autoclaved (S1, S2) and filtered (S3) seawater from the IMTA system. S1 and S3 were kept in climate chamber 1 and, to trigger the conchosporangia formation, S2 was cultured in different conditions at climate chamber 2. Cultures from S3 then proceed to the outdoor system as adult blades S4 (15–1,000 L) and S5 (7–20 m^2^). In these stages, the algae were cultured with filtered (100 μm pore size filter) seawater from the IMTA system, under controlled stocking densities, renovation rates and aeration conditions.

### 2.2 Isolation of *P. dioica*-associated yeasts

Samples from the S1, S2, and S3 stages correspond to early life stages (up to 0.5 cm) and for this reason, before utilization, were only subjected to vigorous shaking. However, the S4 and S5 stages of the macroalgae correspond to more developed life stages with bigger blade dimensions requiring a different approach. In this case, a portion of the blade was separated and cut into smaller fragments (approx. 0.5 cm long) using a sterile scalpel. The fragments were then mixed with 5 mL of the respective culture water and shaken vigorously.

For yeast isolation, samples from macroalgal cultures were directly plated in the media - either directly the algae or the culture water mixed with the macroalgae fragments. In the case of macroalgae mixed with water, the samples were serially diluted (10-fold) in 50% (v/v) Artificial Sea Water [ASW; 23.38 g/L NaCl (PanReact, Barcelona, Spain), 2.41 g/L MgSO_4_·7H_2_O (LabChem, Zelienople, PA, United States), 1.9 g/L MgCl_2_·6H_2_O (Fluka Analytical, Buchs, Switzerland), 1.11 g/L CaCl_2_·2H_2_O (Merck, Darmstadt, Germany), 0.75 g/L KCl (Merck), 0.17 g/L NaHCO_3_ (LabChem) and ddH_2_O]. The original samples and, when adequate, diluted samples, were spread (100 μL) onto YPD-Chl-agar plates and WLN-Chl-agar plates, both supplemented with chloramphenicol (Chl), used to avoid susceptible bacteria growth. The composition of YPD-Chl-agar medium is 10 g/L yeast extract (VWR Chemicals, Radnor, PA, United States), 20 g/L peptone (BD Gibco, Waltham, MA, United States), 20 g/L glucose (Scharau, Barcelona, Spain), 20 g/L agar (LabChem), 100 μg/mL chloramphenicol (Sigma, Burlington, MA, United States) and 50% (v/v) ASW. The composition of WLN-Chl-agar is 4 g/L yeast extract, 5 g/L tryptone (NZYTech, Portugal), 50 g/L glucose, 0.55 g/L KH_2_PO_4_ (Sigma), 0.425 g/L KCl, 0.125 g/L CaCl_2_ (Merk), 0.125 g/L MgSO_4_ (LabChem), 0.022 g/L Bromocresol green (Sigma), 0.0025 g/L FeCl_3_ (ThermoFisher), 0.0025 g/L MnSO_4_ (Sigma), 100 μg/mL chloramphenicol and 50% (v/v) ASW.

Additionally, an enrichment culture was prepared using liquid YPD-Chl medium (the same composition as YPD-Chl-agar medium, without agar) with the sample, algae fragments and culture water, constituting 10% (v/v) of the final volume. The culture was incubated at 22 °C with orbital agitation for up to 72 h. After 24 h and 72 h, the culture and respective 10-fold dilutions were plated onto YPD-Chl-agar and WLN-Chl-agar, as described.

All the plates were incubated at 22 °C for up to 10 days. Following incubation, the plates were observed, and the colonies counted. Colonies of different morphologies, if present, were selected, and cells were observed on an Axioplan microscope (1000×) (Zeiss, Oberkochen, Germany) to differentiate yeast colonies from colonies of chloramphenicol-resistant bacteria. Isolated colonies (two to four colonies with different morphologies, whenever possible) were picked and streaked for purity onto the YPD-Chl-agar medium. For long-term storage, yeast isolates were preserved at −80 °C in their isolation medium containing 15% (v/v) glycerol and included in the IST-Yeast Culture Collection (IST-Yeast CC, Instituto Superior Técnico, Lisbon, Portugal; https://blueyeastscc.tecnico.ulisboa.pt/).

### 2.3 Molecular identification at the species level

Molecular identification of yeast isolates was achieved as described in (Matos et al., 2025a,b). Briefly, genomic DNA was extracted following a method adapted from (Lee et al., 2012). Genomic DNA was used as template to amplify the D1/D2 domain sequence of the 28S ribosomal DNA (rDNA) and the internal transcribed spacer (ITS) region of rDNA. The primer pairs used for D1/D2 and ITS amplification were, respectively, NL-1 (5′-GCATATCAATAAGCGGAGGA AAAG-3′) and NL-4 (5′-GGTCCGTGTTTCAAGACGG-3′), and ITS1 (5′-TCCGTAGGTGAACCTGCGG-3′) and ITS4 (5′-TCCTCCGC TTATTGATATGC-3′), which are considered effective for the taxonomic identification of yeasts (Kurtzman and Robnett, 1998). The yeast species were identified by running a nucleotide BLAST (http://www.ncbi.nlm.nih.gov/blast, assessed in 2024) with the D1/D2 and ITS-obtained sequences against the core nucleotide NCBI database. All D1/D2 and ITS sequences were submitted to GenBank (Table 1).

**Table 1:** Yeast isolates retained from the different culture samples of *Porphyra dioica*.

Samples	ID	Species	NCBI accession number		Isolation conditions
			D1/D2 region	ITS region	
S1	IST650	*Naganishia diffluens*	PP156551	PP158638	Enrichment: WLN-Chl-agar plates inoculated with enrichment culture [YPD-Chl with 50% ASW (72 h)].
	IST651	*Naganishia diffluens*	PP156552	PP158639	Enrichment: WLN-Chl-agar plates inoculated with enrichment culture [YPD-Chl with 50% ASW (72 h)].
	IST652	*Naganishia diffluens*	PP156553	PP158640	Enrichment: WLN-Chl-agar plates inoculated with enrichment culture [YPD-Chl with 50% ASW (72 h)].
	IST653	*Naganishia diffluens*	PP341334	PP341882	Enrichment: WLN-Chl-agar plates inoculated with enrichment culture [YPD-Chl with 50% ASW (72 h)].
S2	IST654	*Rhodotorula mucilaginosa*	PP341335	PP341883	Direct: WLN-Chl-agar plates inoculated with macroalgae sample.
	IST655	*Rhodotorula mucilaginosa*	PP156554	PP158641	Enrichment: YPD-Chl-agar plates inoculated with enrichment culture [YPD-Chl with 50% ASW (24 h)].
	IST656	*Rhodotorula mucilaginosa*	PP341336	PP341884	Enrichment: YPD-Chl-agar plates inoculated with enrichment culture [YPD-Chl with 50% ASW (24 h)].
	IST657	*Rhodotorula mucilaginosa*	PP156555	PP158642	Enrichment: WLN-Chl-agar plates inoculated with enrichment culture [YPD-Chl with 50% ASW (24 h)].
	IST658	*Rhodotorula mucilaginosa*	PP156556	PP158643	Enrichment: YPD-Chl-agar plates inoculated with enrichment culture [YPD-Chl with 50% ASW (72 h)].
	IST659	*Rhodotorula mucilaginosa*	PP156557	PP158644	Enrichment: WLN-Chl-agar plates inoculated with enrichment culture [YPD-Chl with 50% ASW (72 h)].
	IST667	*Rhodotorula mucilaginosa*	PP156558	PP158645	Enrichment: WLN-Chl-agar plates inoculated with enrichment culture [YPD-Chl with 50% ASW (72 h)].
	IST661	*Rhodotorula mucilaginosa*	PP156559	PP158646	Enrichment: YPD-Chl-agar plates inoculated with enrichment culture [YPD-Chl with 50% ASW (72 h)].
S4	IST662	*Williopsis vartiovaarae*	PP156560	PP158647	Enrichment: WLN-Chl-agar plates inoculated with enrichment culture [YPD-Chl with 50% ASW (72 h)].
	IST663	*Williopsis vartiovaarae*	PP341337	PP341885	Enrichment: YPD-Chl-agar plates inoculated with enrichment culture [YPD-Chl with 50% ASW (72 h)].
	IST664	*Williopsis vartiovaarae*	PP156561	PP158648	Enrichment: YPD-Chl-agar plates inoculated with enrichment culture [YPD-Chl with 50% ASW (72 h)].
	IST665	*Williopsis vartiovaarae*	PP156562	PP158649	Enrichment: WLN-Chl-agar plates inoculated with enrichment culture [YPD-Chl with 50% ASW (72 h)].
	IST666	*Williopsis vartiovaarae*	PP341338	PP341886	Enrichment: WLN-Chl-agar plates inoculated with enrichment culture [YPD-Chl with 50% ASW (72 h)].
	IST668	*Williopsis vartiovaarae*	PP156563	PP158650	Enrichment: YPD-Chl-agar plates inoculated with enrichment culture [YPD-Chl with 50% ASW (24 h)].
	IST669	*Taphrina* sp.	PP156564	PP158651	Direct: YPD-Chl-agar plates inoculated with macroalgae sample.
S5	IST671	*Naganishia diffluens*	PP156565	PP158652	Enrichment: WLN-Chl-agar plates inoculated with enrichment culture [YPD-Chl with 50% ASW (24 h)].
	IST724	*Sporobolomyces roseus*	PP156566	PP158653	Enrichment: YPD-Chl-agar plates inoculated with enrichment culture [YPD-Chl with 50% ASW (72 h)].

Information on the isolate ID, isolation conditions, taxonomic species, GenBank NCBI accession number of the D1/D2 and ITS consensus sequences deposited, is provided.

### 2.4 Phylogenetic analysis of yeast isolates

For the phylogenetic placement of the 21 yeast isolates obtained in this work, the D1/D2 consensus rDNA sequences were aligned iteratively with the sequences of the type strains for each species, whose GenBank accession numbers are given on Supplementary Table 1. Multiple alignment was carried out with Muscle, available in the software MEGA v.12.1.2. The Mega software was also used for the phylogenetic tree construction using the maximum likelihood method of the Kimura 2-parameter model, selected based on the MEGA software recommendation considering the data (Tamura et al., 2021). The confidence level of the clades was estimated using bootstrap analysis with 500 replicates. The same protocol was followed for the construction of a phylogenetic tree using the ITS sequences of isolate IST669 and of the type strains of *Taphrina* species indicated in Supplementary Table 1.

### 2.5 Screening the yeast isolates for biotechnological applications

The 20 selected isolates (Table 1, except IST669) were tested for biosurfactant, lipid and auxin production capacity. Additionally, one *Rhodotorula toruloides* strain (IST536 MM15) was used as positive control for lipid intracellular accumulation. This high-producer multistress resistant strain was obtained through Adaptive Laboratory Evolution (ALE) from strain IST536, selected in our lab to produce lipids from sugar beet hydrolysates (Martins et al., 2021).

Yeast cells were pre-cultured in liquid YPD medium (prepared with ddH_2_O) for 24 h with orbital shaking and incubated at 22 °C. Pre-cultured cells were harvested by centrifugation at 4600×g for 5 min at 4 °C and inoculated in 20 mL of minimal medium [6.7 g/L of Yeast Nitrogen Base (BD Difco), 20 g/L of glucose with pH adjusted to 5.5] in 100 mL shake flasks, at an initial optical density at 600 nm (OD600nm) of 1. The cultures were incubated at 22 °C with orbital agitation and growth was monitored by measuring OD600nm using a U-2000 HITACHI spectrophotometer (Tokyo, Japan). This medium was filter sterilized using a 0.2 μm filter (Whatman® Puradisc, Maidstone, United Kingdom).

#### 2.5.1 Biosurfactant production assessment

##### 2.5.1.1 Oil-displacement test

Biosurfactant production, after 144 h of cultivation in a minimal medium with glucose as a carbon source, was assessed with the oil displacement test performed as described before (Sharma et al., 2019). Briefly, 20 mL of water was added to Petri dishes, followed by 1 mL of car oil—(1:46) made of used car oil (to provide color to the mixture) and new car oil (NorAuto ACEA A5/B5 WSS-M2C913-D/5W-30). Finally, 40 μL of cell-free culture supernatant (collected by centrifugation at 3300×g for 3 min) was placed onto the oil drop surface. The diameter of oil displacement was measured, with a ruler, after stabilization of the diameter. The test was performed in triplicate and a 1% SDS solution (w/v) (Sigma) was used as positive control while the cultivation medium was used as negative control.

##### 2.5.1.2 Emulsification index

The emulsification Index was determined as described in (Sharma et al., 2019) after 144 h of cultivation in minimal medium. Briefly, 800 μL of the culture supernatant was added to 800 μL of olive oil (1:1 ratio) in a 2 mL Eppendorf tube and vortexed for 2 min. After 24 h, the total height (cm) of the liquid and the emulsified layer height (cm) were measured. The emulsification index was calculated by dividing the emulsified layer height by the total height and was expressed as a percentage. The test was performed in triplicate and positive and negative controls were those used for the oil-displacement test.

#### 2.5.2 Lipid accumulation assessment

The assessment of lipid accumulation in yeast cells was carried out based on Nile Red staining (Morales-Palomo et al., 2022), routinely used in our lab (Martins et al., 2021; Fernandes et al., 2023). To reach a final OD600nm of 1.5, the cultures that were prepared for biosurfactant assessment were harvested after 50 h of incubation. Cells were collected by centrifugation ( 2400×g , 3 min) and the pellet resuspended and washed twice with a solution of 0.9% (w/v) NaCl and the washed pellets resuspended in Phosphate-Buffered Saline [PBS; 1×, pH 7.4, containing 8 g/L NaCl (Sigma), 0.2 g/L KCl (Sigma), 1.44 g/L Na_2_HPO_4_ (Sigma) and 0.24 g/L KH_2_PO_4_]. These cell suspensions were incubated at 50 °C for 30 min, and a total of 200 μL was transferred to a black 96-well clear bottom plate (Thermo Fisher Scientific), to measure the cell optical density at 600 nm in a POLARstar OPTIMA Microplate Reader (BMG Labtech, Ortenberg, Germany). Cell suspensions were stained with Nile Red (Sigma-Aldrich) at a final concentration of 2.5 μg/mL, and the relative fluorescence units (RFU) were measured using a POLARstar OPTIMA Microplate Reader Software v2.20R2, using the excitation and emission wavelengths of 535 and 625 nm, respectively. To obtain the final values of relative fluorescence units, the background fluorescence values were subtracted: (i) cells with PBS without Nile Red to eliminate buffer autofluorescence, and (ii) PBS with Nile Red. A normalization step of fluorescence to OD600nm was also performed to consider the variation in cell concentration.

#### 2.5.3 Auxin production assessment

The auxin production was assessed under standardized conditions and carried out based on the method with the Salkowski reagent, performed as described before (Gordon and Weber, 1951; Sun et al., 2014). Briefly, after 72 h of cultivation in the minimal medium, 500 μL of supernatant were mixed with 500 μL of Salkowski reagent [2 mL of 0.5 M FeCl_3_ and 98 mL of 35% HClO_4_ (Merck)], and the intensity of the pink color of the mixture, after 30 min of incubation in the dark at room temperature, was quantified with a spectrophotometer at 530 nm. A calibration curve was made and prepared using indole-3-acetic acid (Sigma) as standard.

## 3 Results

### 3.1 Culturable yeasts obtained from *P. dioica* culture samples

Yeast isolation was based on a protocol previously optimized for yeast isolation from microalgae cultures by our team (Matos et al., 2025a,b). Shortly, six YPD-Chl-agar plates and six WLN-Chl-agar plates were inoculated either with the macroalgae fragments, the aqueous part of the culture, or with cultures resulting from enrichment of the original sample in YPD-Chl liquid media. Based on different colony morphologies observed on the plates, two to four colonies were selected and streaked onto YPD-Chl-agar plates. This approach was chosen to preserve the full extent of the potential biological diversity present in each sample, given that phenotypically indistinguishable colonies may correspond to different species, isolates were subsequently subjected to molecular characterization for reliable species-level identification.

**Figure 2:**
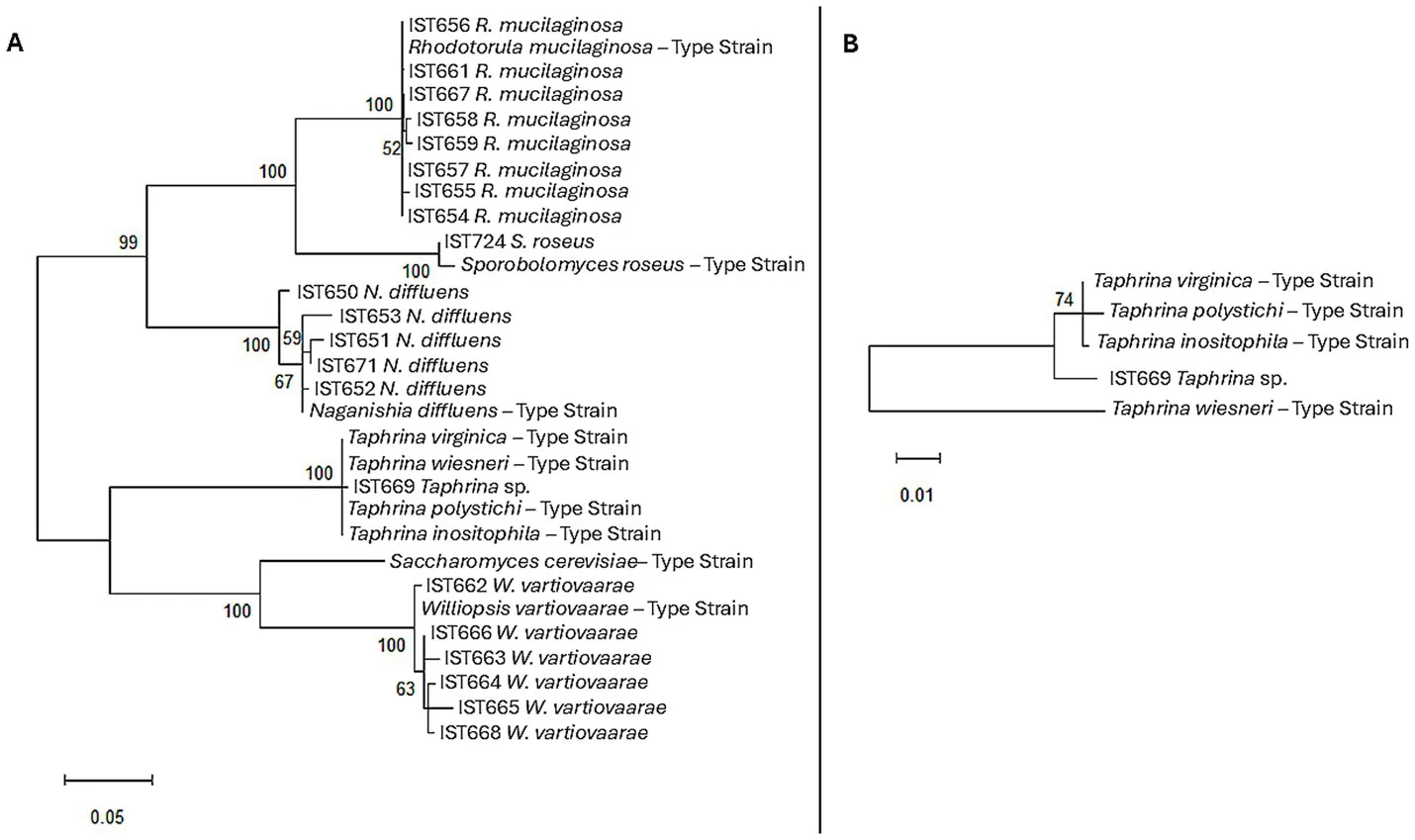
Phylogenetic analysis of the yeast isolates obtained in this study. **(A)** The phylogenetic taxonomic assignment of all isolates was based on the alignment of sequences of the D1/D2 domain of the 28S rDNA region, inferred by means of the maximum likelihood method and Kimura 2-parameter model. **(B)** The phylogenetic taxonomic assignment of IST669 was also based on the alignment of sequences of the ITS domain of the rDNA region, inferred by means of the maximum likelihood method and Kimura 2-parameter model. Sequences from the type strains of the different yeast species were included (Supplementary Table 1). The scale bar indicates the number of expected substitutions per site. The numbers provided at the branches are frequencies in percentage with which a given branch appeared in 500 bootstrap replications.

The subsequent processing of the five samples resulted in the retention of 21 yeast isolates. The 21 yeast isolates retained were molecularly identified (Table 1) based on the comparison of their D1/D2 and ITS nucleotide sequences with the sequences deposited in the NCBI database and their nucleotide sequences were submitted to GenBank. Yeast molecular identification at the species level is consistent with the phylogenetic analysis performed using D1/D2 consensus region and the maximum likelihood inference method, which placed the isolates close to the type strains for each species (Figure 2A).

Most of the isolates were obtained after enrichment in liquid YPD-Chl medium with 50% of ASW, except for IST654 and IST669 which were obtained by direct plating of the macroalgae sample.

All isolates were identified to species level, except *Taphrina* sp. (IST669), which could only be identified to genus level. In this case, the D1/D2 region shared 100% identity with the respective regions of strains from *Taphrina virginica*, *Taphrina wiesneri*, *Taphrina polystichi* or *Taphrina inositophila*, while the ITS region shared an identity of approx. 98.5% with a strain from *Taphrina inositophila* and several uncultured fungi deposited in the database. To better clarify the molecular identification of IST669, a phylogenetic tree (Figure 2B) of the more closely related *Taphrina* species to IST669 was constructed based on the ITS sequences of IST669 and the type strains. No conclusion could be drawn based on the phylogenetic tree. This result are align with the very low interspecific variability in the standard fungal barcode regions reported for the *Taphrina* genus (Rodrigues and Fonseca, 2003). *Taphrina* species are known parasitic plant pathogens in their filamentous state, and their growth on artificial media, although possible, is extremely slow (Rodrigues and Fonseca, 2003; Inácio et al., 2004; Tsai et al., 2014), as observed in our work.

In S1, yeast isolates were only obtained on agar plates inoculated with the enrichment culture. From these plates, four yeast colonies were selected and all identified as *Naganishia diffluens*.

The direct plating of S2 algal fragments resulted in the appearance of some filamentous fungi along with one red yeast colony surrounding the fragments. From the plates inoculated with the enrichment culture, seven isolates were also retained. All isolates retained, either from direct sample or the enrichment culture plating, were molecularly identified as *Rhodotorula mucilaginosa*, strengthening the reliability of the isolation of this species in the sample.

As previously mentioned, *P. dioica* was cultivated with seawater from an IMTA system. However, contrarily to S1 and S2, in which autoclaved water was used, in S3, S4 and S5 the water was not sterilized, only filtered with a 100 μm filter. This change in water disinfection resulted in the appearance of a very high number of chloramphenicol-resistant bacterial colonies, identified by 16S rRNA gene Sanger sequencing as *Pseudomonas* spp. and *Enterobacter* sp. (results not shown), deeply affecting the isolation of yeasts from samples S3 to S5. In fact, no yeasts could be isolated from S3 sample (young blades) plates among the bacteria colonies present.

As referred, S4 and S5 samples, corresponding to adult blades, were also impacted by a high concentration of chloramphenicol-resistant bacteria. Nevertheless, from S4, seven yeast isolates were retained, six *Williopsis vartiovaarae* (formerly known as *Cyberlindnera vartiovaarae* and *Candida vartiovaarae*) isolates, and one *Taphrina* sp. isolate, obtained from the direct inoculation of the sample. Finally, from S5, two isolates, one *Sporobolomyces roseus* and one *N. diffluens*, were obtained from the inoculation of an enrichment culture.

Overall, the 21 yeast isolates belong to five different species, *N. diffluens*, *R. mucilaginosa* and *S. roseus* from the phylum Basidiomycota and *W. vartiovaarae* and *Taphrina* sp. from the phylum Ascomycota.

### 3.2 Production of biotechnologically and ecologically relevant compounds by yeast isolates

**Figure 3:**
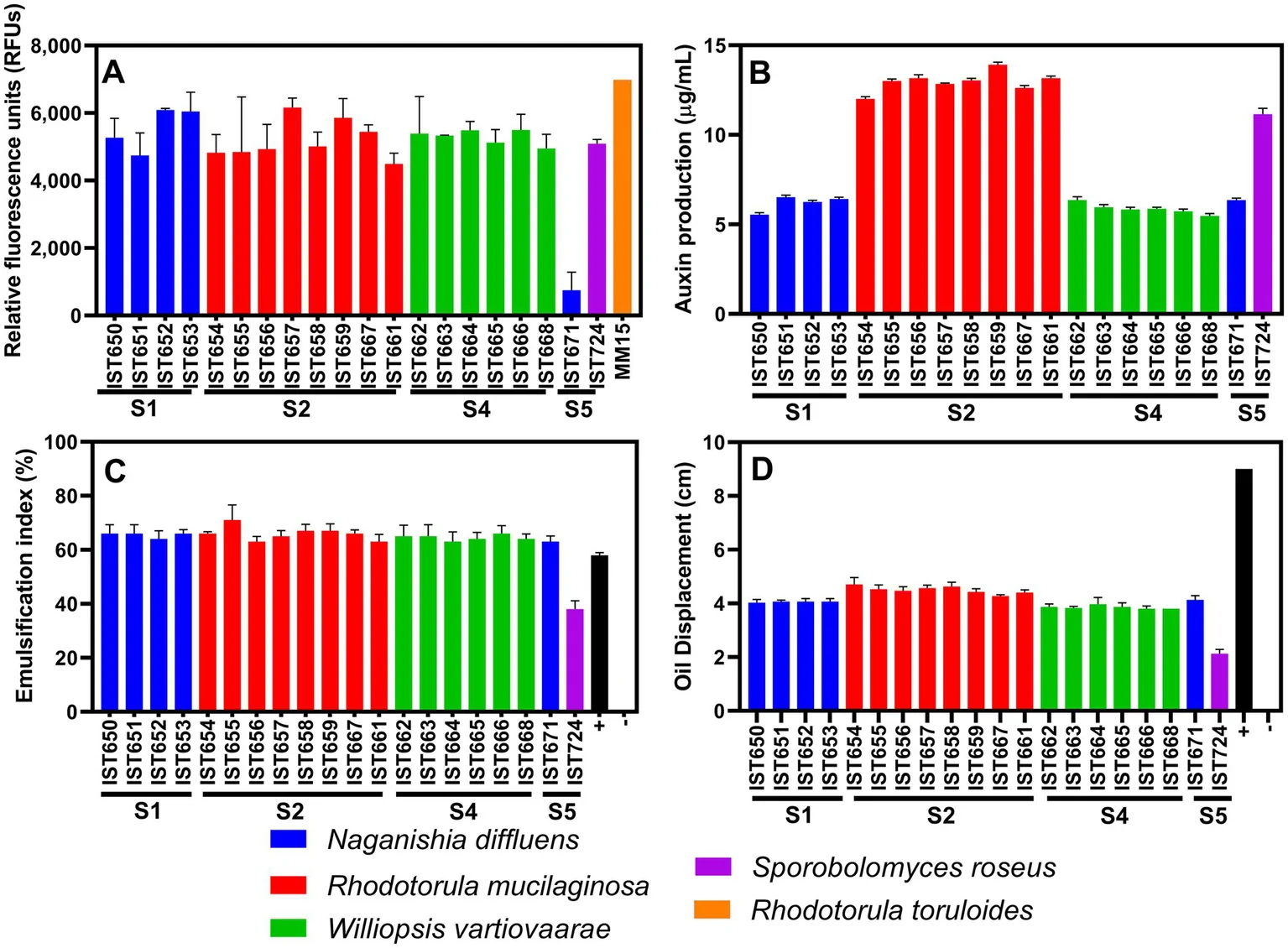
Production of biotechnologically and ecologically relevant compounds by yeasts isolated from different life stages of the cultivated macroalga *Porphyra dioica*. Lipid production **(A)** was assessed by Nile Red staining after 50 h of cultivation and is given in RFU (Relative Fluorescence Units). Auxin production **(B)** was assessed using the Salkowski reagent methods after 72 h of cultivation. Biosurfactant/bioemulsifier production was assessed through the emulsification index **(C)** and the oil displacement test **(D)** after 144 h of growth. The isolates were cultured in a minimal medium with 20 g/L glucose at pH 5.5. For the biosurfactant production assays, positive control was a solution of 1% SDS (w/v) and the negative control was the growth medium. *Rhodorula toruloides* IST536 MM15 (Fernandes et al., 2023) was used as a positive control for lipid production. The data shown represents the average of three independent experiments, and the error bars indicate standard deviation. Details on the identity of the yeast isolates are provided in Table 1. The numerical values of data displayed can be found in Supplementary Table 2.

Several yeasts species can produce important compounds in the context of a circular bioeconomy. *Rhodotorula mucilaginosa* has previously been described as an auxin, lipid and biosurfactant/bioemulsifier producer (Matos et al., 2025a,b). Therefore, in this work, 20 of the 21 yeast isolates obtained from the *P. dioica* culture samples were cultured in a minimal medium with glucose for the assessment of lipids (after 50 h of growth, Figure 3A), auxins (after 72 h of growth, Figure 3B) and biosurfactants/bioemulsifiers (after 144 h of growth, Figures 3C,D). The *Taphrina* sp. isolate (IST669) was not tested since it was impossible to attain consistent growth in laboratory conditions. *Rhodorula toruloides* IST536 MM15 (Fernandes et al., 2023) was used as a positive control for lipid production.

Lipid production (Figure 3A) was assessed through the Nile Red staining method. As observed before (Matos et al., 2025b), the eight *Rhodotorula mucilaginosa* isolates (e.g., IST785) accumulated similarly high contents of intracellular lipids under standardized conditions, although their lipid contents were still lower than those of the positive control. *Naganishia diffluens* and *Sporobolomyces roseus* species were also described as oleaginous yeasts, as corroborated by our results (Ananda and Vadlani, 2010; Li M. et al., 2022). The *N. diffluens* isolate IST671, obtained from the S5 sample, revealed to be a poor lipid producer under the standardized conditions used, markedly different from the *N. diffluens* isolates obtained from S1 sample. All *Williopsis vartiovaarae* isolates were also capable of producing high levels of lipids.

The auxin production test (Figure 3B), carried out through the Salkowski reagent method, revealed that *Rhodotorula mucilaginosa* isolates exhibited the highest auxin production, on average 13 μg/mL, followed by *Sporobolomyces roseus* with 11.2 μg/mL. Although *R. mucilaginosa* IST661 from S5 produced lower levels of lipids compared with *R. mucilaginosa* isolates from S1, all *R. mucilaginosa* isolates produced similar levels of auxins, under the tested conditions.

Biosurfactant/bioemulsifier production was assessed based on the oil displacement test and the emulsification index (Figures 3C,D). All tested isolates produced biosurfactant/bioemulsifier with similar activities, except for *S. roseus* IST724, which showed lower activity.

## 4 Discussion

In the present study, we isolated and characterized the culturable yeast community associated with five life stages of the red macroalga *Porphyra dioica* produced under the Integrated Multi-Trophic Aquaculture (IMTA) concept at ALGAplus. To the best of our knowledge, this represents the first description of the culturable yeast fraction associated with *P. dioica* produced in an IMTA system. In IMTA, nutrient-enriched water from fed aquaculture is reused for the cultivation of extractive organisms such as macroalgae, promoting nutrient recycling and reducing the environmental impact of aquaculture effluents (Hossain et al., 2022; Kong et al., 2023; Pereira et al., 2025). IMTA-derived seawater is typically enriched in dissolved inorganic nitrogen, phosphate, and organic matter (Hossain et al., 2022; Kong et al., 2023). Since *P. dioica* efficiently assimilates nitrogen, particularly during blade development, this species is well suited for biomass production in IMTA systems and may contribute to nutrient bioremediation (Pereira et al., 2008).

### 4.1 Overview of the culturable yeast fraction recovered

The five *P. dioica* cultivation stages examined were produced under different conditions. S1 and S2, corresponding to conchocelis and conchosporangia, were grown indoors using autoclaved IMTA-derived seawater, whereas S3 to S5 were cultivated using filtered, non-sterilized seawater. In addition, S1, S2, and S3 were maintained indoors in photobioreactors, while S4 and S5 were grown in outdoor tanks. These differences are important because microbial communities may be shaped not only by host developmental stage, but also by water treatment, microbial inoculum, irradiance, temperature, hydrodynamics, and organic matter availability. Therefore, the yeast taxonomic distribution observed in this study should be interpreted as the combined outcome of host-, environmental, and cultivation-related factors.

Overall, 21 yeast isolates were retained from the five life stages of *P. dioica*, belonging to *Rhodotorula mucilaginosa*, *Naganishia diffluens*, *Williopsis vartiovaarae*, *Taphrina* sp., and *Sporobolomyces roseus*. The low number of isolates, together with the fact that several were recovered only after enrichment, suggests that yeasts were present at low abundance or were difficult to recover under the culture conditions used. Thus, these results represent the culturable and recoverable yeast fraction rather than the complete yeast community associated with *P. dioica*. This limitation is particularly relevant for S3 to S5, where high numbers of chloramphenicol-resistant bacteria were detected in samples cultivated with filtered, non-sterilized seawater. Bacterial overgrowth likely reduced yeast recovery and may have biased the observed assemblage toward taxa able to tolerate the enrichment and isolation conditions.

### 4.2 Ecological relevance of the recovered yeast taxa

The recovery of *R. mucilaginosa*, *N. diffluens*, and *S. roseus* is consistent with previous reports of pigmented yeasts from marine, estuarine, and aquaculture-associated environments (Fell, 2012; Matos et al., 2025a,b; Tarnecki et al., 2025; Martínez-Rodrigo et al., 2026). These taxa display physiological plasticity and broad substrate use, traits that may be advantageous in aquaculture and estuarine systems characterized by fluctuating nutrient availability, organic matter inputs, salinity variation, irradiance, and oxidative stress (Hu et al., 2025; Tarnecki et al., 2025; Zhao et al., 2025). Their ability to assimilate different inorganic and organic nitrogen sources such as ammonium ions and amino acids, may also help explain their occurrence in an IMTA-derived cultivation environment enriched in dissolved nitrogen forms (Hu et al., 2025; Tarnecki et al., 2025; Zhao et al., 2025). However, whether these yeasts actively contribute to nitrogen transformation at the algal surface remains to be experimentally demonstrated.

### 4.3 Yeast shifts between early life stages S1 and S2

A marked difference was observed between the yeast taxa recovered from S1 and S2. From S1, only *N. diffluens* was isolated, and only after enrichment, whereas from S2 only *R. mucilaginosa* was retrieved, including one isolate obtained by direct plating. Since both stages were cultivated with autoclaved IMTA-derived seawater and no chloramphenicol-resistant bacteria were detected, this difference may reflect physiological changes during the transition from conchocelis to conchosporangia. This transition is known to be influenced by environmental cues such as temperature, light intensity, and photoperiod (Pereira et al., 2004), and may be accompanied by changes in surface chemistry, nutrient exchange, and dissolved organic carbon release. Algal morphogenetic and growth-promoting compounds released by marine microorganisms naturally occur in coastal seawater (Grueneberg et al., 2016) and are known to determine the development of macroalgae at early developmental stages (Egan et al., 2001; Marshall et al., 2006; Spoerner et al., 2012; Grueneberg et al., 2016). Axenic cultures of the closely related *Pyropia yezoensis* have been shown to lose normal morphogenetic capacities compared with individuals harboring a natural microbiome (Yamazaki et al., 1998). Bacterial taxa such as *Maribacter* and *Roseobacter*, commonly associated with macroalgae, have been identified as important contributors to algal growth through the production of morphogenetic compounds such as thallusin and auxin-like phytohormones (Weiss et al., 2017; Alsufyani et al., 2020). While the contribution of yeasts to macroalgal morphogenesis remains poorly understood, yeast-derived indole-3-acetic acid (IAA) has been shown to promote early development in *Zea mays* (maize) (Bispo et al., 2023) suggesting that yeast-produced phytohormones may influence the development of other photosynthetic organisms.

The differential recovery of *N. diffluens* from S1 and *R. mucilaginosa* from S2 is therefore consistent with life-stage-dependent niche differentiation within the algal microbiome. *N. diffluens* is frequently associated with marine or nutrient-variable environments and displays physiological versatility, which may confer an advantage under fluctuating or relatively low-carbon conditions. In contrast, *R. mucilaginosa* is a metabolically versatile, carotenoid-producing yeast commonly reported from aquatic and marine-associated environments (Li Z. et al., 2022). Its recovery from S2 may be compatible with a more metabolically active or photosynthetically responsive stage, where increased availability of algal-derived organic carbon and oxidative stress could favor pigmented, stress-tolerant yeasts. Nevertheless, given the limited number of isolates, this apparent transition should be interpreted cautiously. Additional culture-independent profiling would be required to determine whether this pattern reflects a true community-level shift. Indeed, community-level shifts have been investigated using both cultivation-independent and cultivation-dependent methods in the prokaryotic communities associated with *P. dioica* during the S1–S3 stages cultivated at ALGAplus (Cortez et al., 2026). The structure of *Porphyra*-associated prokaryotic communities shifted significantly across the host’s developmental stages, with a relative enrichment of *Alphaproteobacteria* and *Bacteroidota* in S1 and S2, followed by their strong reduction in S3, accompanied by a marked increase in *Gammaproteobacteria*, mainly of the family *Nitrinicolaceae* (valid synonym: *Oceanospirillaceae*), in stage S3. Clear shifts between early developmental stages of *Porphyra* were also observed in the cultured bacterial fraction, thus corroborating the findings reported here for yeasts.

### 4.4 Transition from Ascomycetous to Basidiomycetous yeasts between S4 and S5

A second relevant shift was observed between S4 and S5. The S4 stage yielded *W. vartiovaarae* and *Taphrina* sp., whereas S5 yielded *N. diffluens* and *S. roseus*. This shift from Ascomycetous yeasts to Basidiomycetous taxa is consistent with ecological succession during blade maturation. S4 may represent a metabolically active and nutrient-enriched stage, favoring opportunistic or carbon-responsive Ascomycetes. Species of *Cyberlindnera,* previous classification of *W. vartiovaarae* are generally known for efficient sugar assimilation and fermentative or facultatively fermentative metabolism (Sousa-Silva et al., 2021), traits that may be advantageous in microenvironments enriched in algal exudates or dissolved organic carbon. However, because little is known about *W. vartiovaarae* specifically, its ecological role in association with *P. dioica* remains uncertain.

The genus *Taphrina* is commonly associated with plants and includes several plant pathogenic species (Rodrigues and Fonseca, 2003; Tsai et al., 2014). However, there is currently no evidence that *Taphrina* causes disease in *P. dioica* or other macroalgae. In this study, the isolate was identified only to genus level due to close molecular similarity to multiple described species. Its presence may therefore not necessarily reflect transient surface colonization, association with organic particles, or an opportunistic interaction with algal tissue during a specific physiological stage. The isolation of some *Taphrina* species, such as *T. inositophila*, from asymptomatic plant material further supports the idea that not all members of this genus are necessarily pathogenic in every host context (Inácio et al., 2004). The difficulty in identifying IST669 at the species level is likely due a very low interspecific variability in the standard fungal barcode regions (ITS and D1/D2 of the LSU rRNA gene) within the *Taphrina* genus (Rodrigues and Fonseca, 2003). To achieve a more robust species-level identification of IST669, the following complementary approaches could be applied in future work: multilocus sequence analysis (MLSA); whole-genome sequencing (WGS) followed by phylogenomic analysis and calculation of overall genome relatedness indices; detailed phenotypic and physiological characterization, including growth on differential media, temperature and pH tolerance, and carbon/nitrogen assimilation profiles, compared with type strains of the closest *Taphrina* species.

In contrast, S5 was associated with *N. diffluens* and *S. roseus*, both Basidiomycetous yeasts with traits commonly linked to environmental persistence. The S5 stage corresponds to adult biomass close to harvest, representing the final phase of the cultivation cycle. At this stage, algal tissues have experienced prolonged exposure to outdoor tank conditions, including variable irradiance, oxidative stress, nutrient fluctuations, and microbial immigration from filtered seawater. Pigmented yeasts such as *S. roseus* produce carotenoids, which may contribute to tolerance against oxidative stress and high light exposure (Moliné et al., 2012; Sandmann, 2022). Similarly, *N. diffluens* is physiologically versatile and may persist under nutrient-variable or oligotrophic conditions. The presence of these taxa in S5 may therefore reflect a shift toward stress-tolerant and environmentally persistent yeasts during the later stages of blade cultivation.

### 4.5 An integrated model of the *P. dioica* culturable mycobiome

Collectively, the yeast distribution observed across *P. dioica* life stages supports a model in which algal developmental physiology interacts with cultivation conditions to shape the culturable mycobiome. Early stages cultivated in autoclaved IMTA-derived seawater showed low yeast recovery and stage-specific isolation of either *N. diffluens* or *R. mucilaginosa*. In adult blade stages cultivated outdoors with filtered seawater, the assemblage shifted from Ascomycetous taxa in S4 to pigmented Basidiomycetous taxa in S5. This pattern is consistent with ecological filtering driven by changes in algal physiology, nutrient availability, organic carbon release, oxidative conditions, and microbial exposure. However, because life stage, water treatment, and cultivation environment were not fully independent variables in the production system, and their relative contributions were not assessed separately, their respective effects and potential interactions cannot be disentangled in the present study. An earlier study focusing on the prokaryotic communities associated with *P. dioica* using cultivation-independent amplicon sequencing, and which assessed algal tissue and surrounding water communities separately during the early life stages (S1–S3), identified differences in the relative abundance of prokaryotic taxa between algal and corresponding water samples. This suggests the establishment of a distinct microenvironment on the alga surface (i.e., phycosphere) which promotes the development of a microbiome that differs from that of the surrounding seawater (Cortez et al., 2026).

The potential functional implications of these yeasts are relevant for aquaculture sustainability. Carbon-responsive and nitrogen-assimilating yeasts may participate in localized nutrient transformation at the algal surface, while stress-tolerant pigmented yeasts may contribute to biofilm stability or persistence under outdoor cultivation conditions. Conversely, shifts in yeast composition may also reflect environmental exposure, substrate availability, or microbial succession without direct functional benefit to the host.

The relatively high lipid content observed in yeasts isolated from the *P. dioica* S1–S4 stages, nearly approaching that of the selected oleaginous yeast positive control, suggests that these macroalgae-associated yeasts possess a notable capacity for intracellular lipid accumulation. As storage lipids represent an important reserve of carbon and energy in oleaginous microorganisms (Abeln and Chuck, 2021), these yeasts may contribute to carbon turnover and storage within the phycosphere surrounding the macroalgal surface. From an applied perspective, the presence of naturally lipid-rich yeasts on macroalgae may have the potential to enhance the nutritional value of the algal biomass, which is of interest for food, feed, and other biotechnological applications (Sá-Correia et al., 2026).

All yeasts tested in this study could produce auxin, with *Rhodotorula mucilaginosa* exhibiting the highest concentrations. The positive effects of yeast-derived auxin on early growth and development have been demonstrated in agricultural crop plants (Kachalkin et al., 2022; Bispo et al., 2023) and endophytic isolates of *R. mucilaginosa* were reported among the more prolific auxin producers identified by Kachalkin et al. (2022). Moreover, auxin biosynthesis was recently reported for *R. mucilaginosa* and *R. sphaerocarpa* isolates associated with the microalga *Tisochrysis lutea* in industrial settings (Matos et al., 2025a). It is therefore plausible to hypothesize that yeast-derived phytohormones may contribute to developmental processes in macroalgae.

Biosurfactants and bioemulsifiers can influence biofilm architecture, regulating microbial attachment and detachment (Ron and Rosenberg, 2001). In this study, all tested yeasts produced biosurfactants and/or bioemulsifiers. This activity may play a regulatory role in the development of biofilms and biofouling on macroalgal surfaces in commercial production systems, where epiphytic overgrowth by microalgae and other fouling microorganisms is a frequent problem (Kim et al., 2014; Patil et al., 2024). Epiphytic outbreaks, including diatom felt or blooms, can reduce thallus growth through shading and nutrient depletion, or cause blade bleaching (Kim et al., 2014; Patil et al., 2024) and present significant challenges for *Porphyra* cultivation, particularly in organic farming systems practiced by companies such as ALGAplus, where no herbicides or pesticides are used. Consequently, biocontrol strategies that leverage microbiome engineering approaches to prevent epiphyte overgrowth and biofilm formation are in high demand.

Overall, isolates assigned to the same species exhibited comparable profiles of lipid accumulation, auxin (IAA) production, and biosurfactant/bioemulsifier activity, as observed for the multiple isolates of *R. mucilaginosa* and *W. vartiovaarae*. This intraspecific consistency is in line with the expectation that conspecific strains share core metabolic traits and further supports the reliability of the molecular identification. A clear exception was found for the two isolates of *Naganishia diffluens*, which differed appreciably in lipid production. Such strain-level variability is not uncommon among environmental yeasts and may reflect genetic or physiological adaptation to distinct microhabitats within the sampling site (Matos et al., 2025a,b). This observation reinforces the relevance of retaining multiple isolates per species during biodiversity surveys, as species-level identification alone may not capture the full functional diversity of the community.

It should be noted that the present screening was intended as an exploratory assessment of functional traits rather than a quantitative ranking of producer strains. The results should therefore be interpreted as indicative of the presence and approximate magnitude of each activity, with dedicated follow-up studies required to fully evaluate the biotechnological potential of the most promising isolates.

### 4.6 Concluding remarks and future perspectives

Future studies combining culture-independent community profiling, quantitative approaches, and physiological assays in *in vivo* macroalgae-yeast co-cultures will be necessary to determine whether these yeasts influence nutrient remediation, pathogen or epiphyte exclusion, algal growth and development, or stress tolerance in IMTA-cultivated *P. dioica*. In summary, this study provides a first characterization of the culturable yeast fraction associated with different life stages of industrially cultivated *P. dioica*. Although the low number of samples and isolates and the influence of cultivation conditions limit broad ecological inference, the stage-associated recovery of distinct yeast taxa suggests that the *P. dioica* mycobiome is dynamic across the life cycle. These findings provide a basis for future work on the functional role of yeasts in macroalgal aquaculture and their possible contribution to the performance and stability of IMTA system. In addition, the screening of the yeast isolates for lipid, biosurfactant and bioemulsifier production carried out in this work reveals that these capacities are common features and highlights their promising biotechnological potential (Matos et al., 2025a,b; Sá-Correia et al., 2026).

## Data Availability

The datasets presented in this study can be found in online repositories. The names of the repositories and accession numbers can be found in the article/Supplementary material.
